# Conversion from twice- to once-daily tacrolimus in pediatric kidney recipients: a pharmacokinetic and bioequivalence study

**DOI:** 10.1007/s00467-013-2724-0

**Published:** 2014-01-17

**Authors:** Anne-Laure Lapeyraque, Nastya Kassir, Yves Théorêt, Maja Krajinovic, Marie-José Clermont, Catherine Litalien, Véronique Phan

**Affiliations:** 1 Service de Néphrologie, Département de Pédiatrie, CHU de Sainte-Justine, Université de Montréal, Montréal, Canada; 2 Unité de Pharmacologie Clinique, CHU de Sainte-Justine, Université de Montréal, Montréal, Canada; 3 Département de Pharmacie, Université de Montréal, Montréal, Canada

**Keywords:** Tacrolimus, Pediatric kidney transplantation, Pharmacokinetic, Pharmacogenetic

## Abstract

**Background:**

The objectives of this study were to investigate pharmacokinetic and pharmacogenetic parameters during the conversion on a 1:1 (mg:mg) basis from a twice-daily (Prograf) to once-daily (Advagraf) tacrolimus formulation in pediatric kidney transplant recipients.

**Methods:**

Twenty-four-hour pharmacokinetic profiles were analyzed before and after conversion in 19 stable renal transplant recipients (age 7–19 years). Tacrolimus pharmacokinetic parameters [area under the concentration-time curve (AUC_0–24_), minimum whole-blood concentration (C_min_), maximum whole-blood concentration (C_max_), and time to achieve maximum whole-blood concentration (t_max_)] were compared between Tac formulations and between CYP3A5 and MDR1 genotypes after dose normalization.

**Results:**

Both AUC_0–24_ and C_min_ decreased after conversion (223.3 to 197.5 ng.h/ml and 6.5 to 5.6 ng/ml; *p* = 0.03 and 0.01, respectively). However, the ratio of the least square means (LSM) for AUC_0–24_ was 90.8 %, with 90 % CI limits of 85.3 to 96.7 %, falling within bioequivalence limits. The CYP3A5 genotype influences the dose-normalized C_min_ with the twice-daily formulation only.

**Conclusions:**

Both tacrolimus formulations are bioequivalent in pediatric renal recipients. However, we observed a decrease in AUC_0–24_ and C_min_ after the conversion, requiring close pharmacokinetic monitoring during the conversion period.

## Introduction

In pediatric kidney transplant recipients, non-compliance with immunosuppressive medications ranges from 5 to 80 % in adolescents [1–3], contributing to late acute transplant rejection and resulting in a 50 % incidence of graft loss [4]. Forgetfulness is the most common reason for non-compliance as reported by caregivers and patients [5]. Compliance is higher with once-daily compared to twice-daily treatment regimens in chronic diseases [6]. Assessment of tacrolimus (Tac) levels is required in clinical practice, because of the narrow therapeutic index and variance in pharmacokinetics (PK) among different patients [7–9].

Advagraf (Astellas Pharma Canada, Inc; Markham, ON, Canada; hereafter referred to as Tac-QD) is a once-daily extended-release formulation of Tac initially developed to improve patient adherence. Clinical trials in stable and de novo solid-organ adult recipients showed similar efficacy, tolerance, and safety when compared to Prograf (Astellas Pharma Canada, Inc; hereafter referred to as Tac-BID) the original twice-daily Tac formulation [10]. Both formulations were shown to be bioequivalent on a 1:1 basis according to the FDA criteria. However, more recent reports indicated that the use of Tac-QD may be associated with a lower Tac exposure (lower C_min_ and lower AUC_0−24_) after a 1:1 conversion from Tac-BID [10–15].

Tac PK parameters have high variability among patients, depending on several factors, such as type of organ transplanted and pharmacogenetics. It is well established that CYP3A5 expression contributes significantly to the variability in Tac PK. Only individuals with at least one CYP3A5*1 allele express a significant amount of CYP3A5 enzyme. The presence of a single-nucleotide polymorphism (SNP) in intron 3 of *CYP3A5* causes alternative splicing and protein truncation resulting in the absence of CYP3A5 enzyme in homozygous carriers (CYP3A5*3/*3) [16–19]. Another important factor affecting the PK of Tac is *MDR1* expression, the gene encoding the active transporter P-glycoprotein [20]. Homozygous individuals for the T-allele in *MDR1* of exon 26 (C3435T), have significantly lower intestinal and leucocyte protein expression than the homozygote C-allele. Other polymorphisms in exon 12 (C1236T) and exon 21 (G2677T) have been studied in Tac PK parameters, and their role remains controversial [16, 21, 22]. Given that the drug release rate and location differ between Tac-BID and Tac-QD, the effect of *CYP3A5* and *MDR1* genotypes on Tac PK parameters may differ between formulations [23].

Therefore, the aims of this study were to compare Tac PK parameters and the impact of *CYP3A5* and *MDR1* genotypes on Tac exposure before and after formulation conversion in stable pediatric renal transplant recipients.

## Materials and methods

This open-label, single-center, PK study was conducted at the Centre Hospitalier Universitaire Sainte-Justine (Montreal, Canada). Health Canada and our Institutional Review Board approved the protocol. The first patient was enrolled on June 29, 2010. Informed consent was obtained prior to participation.

### Patients

Eligible patients were required to be (1) kidney transplant recipients between 6 and 20 years old (able to swallow intact capsules), (2) at least 6 months after transplantation, and (3) taking Tac-BID for at least 2 weeks prior to study entry, in addition to mycophenolic acid and prednisone. Patients were included if their kidney function was stable (no modification in the Tac-BID, mycophenolate mofetil, and steroid doses within 2 weeks prior to enrollment), as well as their hepatic function and general medical condition. Patients were excluded if they (1) were receiving drugs known to interact with Tac metabolism, (2) had begun any new medication within 30 days prior to study enrollment, (3) had had a rejection episode within 180 days before study enrollment, (4) could not swallow capsules, or (5) were receiving rapamycin.

### Study design

Patients were admitted to the Clinical Research facility on the morning of day 1, after having fasted from midnight the day before (day 0) until 60 min after the start of the study. A 24-h PK profile was obtained before conversion (baseline, day 1). Patients were converted to Tac-QD on a 1:1 (mg:mg) basis for their total daily dose on the morning of day 2, and were then discharged from the hospital. Blood samples for the second 24-h PK profile were collected any morning between day 14 and day 42. Serial whole-blood samples were collected immediately before drug administration (pre-dose), and 0.5, 1, 2, 3, 6, 8, 12, 13, 14, 15, 18, 20, and 24 h after.

All immunosuppressants used in combination with Tac were maintained at constant doses until the second 24-h PK profile was performed.

### Pharmacokinetic analysis

Whole blood samples for PK analysis were frozen at −80 °C until analysis then determined using a validated HPLC/MS/MS assay (lower limit of quantification 0.1 ng/ml). AUC were obtained using the linear trapezoidal method applied to the full PK profiles (0 to 24 h). C_min_ values were determined using the observed Tac whole-blood concentration value at the 24-h time point. C_max_ and t_max_ were determined after the morning dose of Tac-BID.

Consistent with the two one-sided test for bioequivalence (Schuirmann, 1987), 90 % confidence intervals (CI) for the ratio between drug formulation least-squares means (LSM) for the Tac-BID to the reference formulation Tac-QD were calculated for the parameters AUC_0−24_ and C_min_ using ln-transformed data and then back transformed to the original scale. The LS means and CI were expressed as a percentage relative to the LS mean of the reference formulation. Tac-BID was considered bioequivalent to Tac-QD if the 90 % confidence intervals (CI) for the LSM ratio fell within the equivalence limits of 80–125 %.

### Genotyping assay

The analyses were performed for three single-nucleotide polymorphisms (SNPs) in the MDR1 gene (1236C/T, 2677 G/AT, 3435C/T) and the CYP3A5 6986 A/G substitution, defining allele *1 and *3, respectively. DNA segments containing the polymorphic MDR1 and CYP3A5 sites were amplified by PCR. Genotyping was performed by allele-specific oligonucleotide (ASO) hybridization, as previously described [24]. Primers set as described by Dulucq and colleagues were used [25].

### Statistical analysis

The clinical characteristics of renal transplant recipients and the PK parameters of Tac-BID and Tac-QD were expressed as the median [range, standard deviations and coefficient of variation (%)]. The Wilcoxon test (paired *t* test) was used to compare Tac PK parameters according to Tac formulations and the Mann–Whitney test was used to compare Tac PK parameters according to CYP3A5 genotype. A *p* value of less than 0.05 was considered statistically significant.

All statistical analysis were made using GraphPad Prism version 5.00 for Windows, GraphPad Software, San Diego, CA, USA.

## Results

### Patient demographics

Patient characteristics are presented in Table 1. Nineteen patients (12 males) between 7 and 18.9 (median age, 15.3) years were included. Median posttransplant duration was 43.7 months (range, 9.5–128.5 months). The median total daily baseline Tac dose was 0.11 mg/kg (0.06–0.19). The allele frequencies of CYP3A5*1/*1, *1/*3, and *3/*3 were 5.3, 21, and 73.7 %, respectively. The alleles of different *MDR1* polymorphisms are summarized in Table 1.

**Table 1:** Clinical characteristics of pediatric renal recipients converting from Tac-BID (Prograf) and Tac-QD (Advagraf)

Characteristics	*n*
Sex	
Male/female	12/7
Race	
Caucasian/black	17/2
Age (years)	15.3 (7–18.9)
Post transplantation time (months)	43.7 (9.5–128.5)
Type of current transplant	
Deceased/living donor	15/4
Previous history of acute rejection	
No/Yes	15/4
Retransplant	
No/Yes	19/0
Total Tac daily dose (mg/kg)	0.11 (0.06–0.19)
Pre-existing non-compliance	
No/Yes	17/2
CYP3A5 genotypes	
*1/*1	1
*1/*3	4
*3/*3	14
MDR1 polymorphisms	
3435C >T	
C/C	4
C/T	10
T/T	5
1236C >T	
C/C	3
C/T	10
T/T	6
2677G >A/T	
G/G	5
G/T	8
TT	6

Values are expressed as the number (*n*) or median (range)

*Tac* tacrolimus

### Tac exposure and PK analysis

Thirty-eight 24-h Tac PK profiles were obtained for 19 patients. The Tac-BID and Tac-QD PK parameters are shown in Table 2.

**Table 2:** Tacrolimus (Tac) pharmacokinetic parameters for Tac-BID (Prograf) and Tac-QD (Advagraf): all patients and CYP3A5 genotypes subgroups

Parameters	Tac-BID (Prograf)				Tac-QD (Advagraf)				
	Median	Range	SD	CV	Median	Range	SD	CV	p*
AUC_0−24_	223.7	149.7–278.6	32.5	15	197.5	129.3–278.5	47.1	23.5	**0.04**
AUC_0−24_/dose	1,815	1,028–4,643	967.6	44.5	1,665	982.7–4,641	1,065	52.3	**0.04**
*1/*1 and *1/*3	1,480	1,177–1,815	288.4	19.5	1,329	982.7–1,665	244.3	18.2	0.31
*3/*3	2,428	1,028–4,643	1,010	41.7	2,111	994.2–4,641	1,140	49.8	0.13
**p**	**0.07**				0.1				
C_min_	6.5	5–8.2	0.8	12.5	5.6	3.5–8.3	1.4	25.2	**0.01**
C_min_/daily dose	55.6	31–125	27.2	41.8	46.	25.3–138.3	30.7	53.1	**0.007**
*1/*1 and *1/*3	43.1	33.2–58.6	10	23.1	37.1	25.3–49.4	8.7	23.2	0.3
*3/*3	75.6	31–125	27.3	37.5	58.3	27.7–138.3	32.7	50.3	**0.01**
**p**	**0.03**				0.1				
C_max_	15.1	11.1–32.6	5.8	33.8	16.3	8.0–28.9	5.4	35.2	0.32
C_max_/daily dose	151.8	61.6–433.3	89.6	53.6	114.3	65–313.3	83.4	53.8	0.32
*1/*1 and *1/*3	145	86.9–182.9	42.3	30.8	90	87.9–180.6	39.9	36.1	0.81
*3/*3	158.1	61.6–433.3	100.5	56.5	159.4	65–313.3	90.1	52.7	0.58
**p**	0.54				0.28				
t_max_	1	1–3			2	1–3			**0.04**

Values are expressed as the median (range). *CV* coefficient of variation (%), *AUC*0–24h, area under the blood concentration-time curve from 0 to 24 h ((ng.h/ml), *Cmin* trough blood concentration at 24 h (ng/ml), *Cmax* maximum blood concentrations (ng/ml), tmax, observed time to reach the maximum blood concentration (h); *1 and *1/*3, CYP3A5 expresser; *3/*3, CYP3A5 nonexpresser; *p* *1 and *1/*3 vs. *3/*3; p*, Tac-BID (Prograf) vs. Tac-QD (Advagraf)

*CV* coefficient of variation, *SD* standard deviation, *AUC*_*0*–*24h*_ 0–24 h area under the tacrolimus concentration-time curve (ng.h/ml), *Dose* total daily tacrolimus dose/weight (mg/kg), *C*_*min*_ minimum whole-blood tacrolimus concentration (ng/ml), *C*_*max*_ maximum whole-blood tacrolimus concentration (ng/ml), *t*_*max*_ time to achieve maximum whole-blood tacrolimus concentration (h), *1 and *1/*3 CYP3A5 expresser, *3/*3 CYP3A5 nonexpresser, *p* *1 and *1/*3 vs. *3/*3, P* Tac-BID (Prograf) vs. Tac-QD (Advagraf)

The median Tac AUC_0−24_ (ng.h/ml) of Tac-BID and Tac-QD was 223.3 and 197.5 (*p* = 0.03), respectively. Despite this statistical difference in AUC_0−24_, the ratio of the least square means (LSM) for AUC_0–24_ was 90.8 %, with 90 % CI limits of 85.3–96.7 % (Table 3), falling within 80 % to 125 % bioequivalence limits. Therefore, the two formulations were bioequivalent.

**Table 3:** Bioequivalence statistics for AUC _0–24h_ and C_min_ for Tac-BID (Prograf) and Tac-QD (Advagraf)

PK	Geometric LSM		Ratio A/B of LSM (%)	90 % CI for ratio of the LSM (%)
Parameters	Tac-QD	Tac-BID		
AUC_0-24_	5.27	5.37	90.82	85.27, 96.73
C_min_	1.68	1.93	77.69	69.33, 87.05

*CI* confidence interval, *PK* pharmacokinetic, *LSM* least square means, *AUC*_*0–24h*_ area under the blood concentration-time curve from 0 to 24 h, *C*_*min*_ trough blood concentration at 24 h

The median C_min_ of Tac-BID (6.5 ng/ml) was significantly higher than Tac-QD median C_min_ (5.6 ng/ml) with a p of 0.01. Furthermore, the ratio of the LSM for C_min_ (77.69 %) and its 90 % CI (69.3–87 %) did not achieve bioequivalence limits of 80–125 % (Table 3). Based on the latter C_min_ results, Tac-BID and Tac-QD are no longer deemed bioequivalent on a 1:1 conversion basis. In addition, no differences were found in C_max_ between formulations. As expected, the observed t_max_ (0 to 12 h) was significantly increased after conversion (1 and 2 h for Tac-BID and Tac-QD, respectively).

**Fig. 1:**
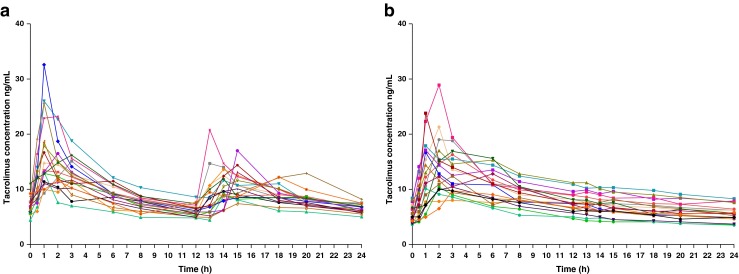
Whole-blood tacrolimus concentration-time profiles in 19 stable pediatric kidney transplant recipients on Prograf (before the conversion) (**a**) and Advagraf (after the conversion) (**b**)

**Fig. 2:**
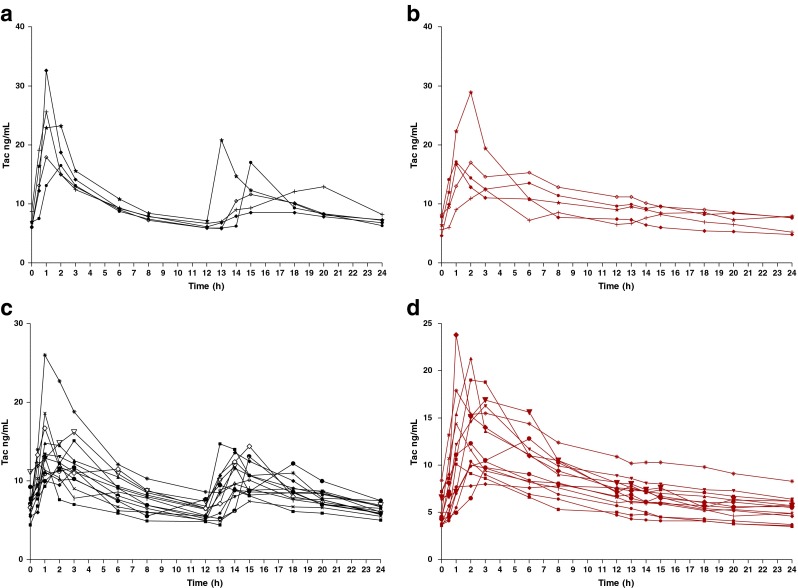
Whole-blood tacrolimus concentration-time profiles in the 5 *CYP3A5* expressers pediatric kidney transplant recipients on Prograf (**a**) and on Advagraf (**b**) and in the 14 *CYP3A5* non-expressers on Prograf (**c**) and on Advagraf (**d**)

The whole-blood Tac concentration-time profiles of the 19 patients are shown in Figs. 1 and 2. We observed high inter-patient variability for the two Tac formulations. Coefficients of variations (CV) for each dose-normalized Tac PK parameters (AUC_0–24h_, C_min_, C_max_) are summarized in Table 2.

### Pharmacogenetic analysis

No association was found between the concentration/dose ratio and *MDR1* genotypes for either Tac formulation.

The CYP3A5 expresser group (*1*1 or 1*3 genotypes) demonstrated lower inter-patient variability (19.5, 23.1, and 30.8 %, respectively) compared to the CYP3A5 nonexpresser group (41.7, 37.5, and 56.5 %, respectively) for all dose-normalized Tac PK parameters (AUC_0–24_, C_min_ and C_max_).

The median dose-normalized C_min_ levels increased in CYP3A5 nonexpressers (*3*3 genotype) compared to expressers with Tac-BID, but not with Tac-QD despite a similar trend (Table 2). Furthermore, median dose-normalized C_min_ levels decreased significantly with Tac-QD compared to Tac-BID in CYP3A5 nonexpressers only.

On the other hand, there were no significant differences in the dose-normalized AUC_0–24h_ between CYP3A5 expressers and nonexpressers with the two formulations, and median dose-normalized AUC_0–24_ was not significantly different between formulations in each group of the *CYP3A5* genotype. Median dose-normalized C_max_ levels were not statistically different between the two formulations and between *CYP3A5* genotypes.

## Discussion

Adolescents are particularly at risk of graft loss because of non-compliance with immunosuppression [26]. Any drug regimen that improves adherence by simplifying its administration is encouraged, although few studies have shown improved adherence one year after conversion to once-daily formulations [27, 28]. The FDA considers Tac-QD, a new formulation of tacrolimus, to be bioequivalent to Tac-BID in adult renal and hepatic transplant recipients [10].

To confirm its bioequivalence in pediatric renal recipients, and to evaluate the safety of conversion, we performed Tac PK studies before and after a 1:1 conversion. The best marker of Tac exposure is the AUC_0–24_, so we assessed 24-h PK profiles before and after conversion for each patient.

In this study, the ratio of the least square means (LSM) for AUC_0–24_ and the 90 % CI limits (Table 3) fell within bioequivalence limits as defined by the FDA. However, we found the 1:1 conversion to be associated with a sustained decrease in Tac exposure, as shown by lower AUC and lower C_min_ (Table 2). Even though the interval between PK profiles was between 14 and 42 days, there were no changes in the patient condition or medications that could have modify Tac pharmacokinetic. Our results are in accordance with recent data reporting Tac-QD to be associated with a significantly lower Tac exposure after a 1:1 conversion in de novo or stable renal and liver transplant recipients [12, 14, 15, 23, 29–40].

Tac is known to have a narrow therapeutic index, already making it tedious to monitor in transplanted patients [41]. An unexpected decrease in Tac exposure may either increase the risk of acute rejection, or conversely cause fewer side effects such as hypertension, hyperglycemia, and nephrotoxicity. An increase in acute rejection has not yet been reported, but the long-term effects of this unexpected decrease in Tac exposure remain unknown. The absence of acute events does not preclude subclinical graft rejection, which may compromise long-term graft survival. The decrease in nephrotoxicity was reported in non-randomized studies [29, 42] but not been confirmed in randomized control trials [43, 44].

These PK results illustrate the increasing evidence that narrow therapeutic index immunosuppressive drugs should not just fulfill standard criteria of bioequivalence [45]. This concern is particularly important in the development of generics [46]. For this reason, the European Medicines Agency and Health Canada recently changed the interval of the relative mean AUC so it would fall within 90–112 % for all drugs inclusively, with a narrow therapeutic index [11]. With these more stringent limits, Tac-QD and Tac-BID may no longer be considered bioequivalent. Therefore, because of the decrease in Tac exposure with Tac-QD, we recommend that pediatric patients should be closely monitored posttransplant. Furthermore, in non-compliant patients, missing one dose may have greater consequences with a single compared to a twice-daily regimen. Furthermore, the impact of Tac-QD on the simultaneous intake of mycophenolic acid (administered twice daily) also needs to be addressed. Taking a single dose of Tac in the morning might increase the risk of the mycophenolic acid evening dose being forgotten. Long-term studies are required to measure adherence of all immunosuppressive medications in this setting.

In contrast to other Tac-QD PK studies in healthy adults and adult kidney transplant recipients, C_max_ did not significantly differ between Tac formulations in our population. On the other hand, as expected, t_max_ was later for Tac-QD, which was absorbed with delay. This element should be monitored closely if a drug interaction is expected to affect the absorption phase of metabolism.

Few studies have compared inter-patient PK variability for Tac-BID and Tac-QD [47]. In our study, we report a moderately higher inter-patient variability in dose-normalized Tac PK parameters (AUC_0–24_ and C_min_) for Tac-QD compared with Tac-BID, with a similar magnitude to that which was reported previously with Tac-BID [48]. Other factors affecting drug absorption (age, ethnicity, gastrointestinal mobility, evening food intake) may explain those discrepancies.

The correlation between Tac C_min_ and *CYP3A5* genotypes also differed between the formulations. Higher dose-normalized C_min_ levels were seen in CYP3A5 nonexpressers (*3*3 genotype) compared to expressers (*1*1 and *1*3 genotypes combined) with Tac-BID, but not with Tac-QD, despite a similar trend. Although differences in dose-normalized AUC in CYP3A5 expresser and non-expressers do not reach the statistical significance the trend is similar to Cmin. Obviously numbers limits the power of the comparisons.

The impact of the genotype of nonexpressers (patients with lower clearance) on the dose-normalized Tac C_min_ is therefore less significant with Tac-QD than with Tac-BID. Furthermore, a notable decrease in dose-normalized C_min_ was observed between formulations only in the CYP3A5 nonexpressers group. These results are consistent with another study in stable adult renal transplant recipients [49]. There is some evidence to suggest that CYP3A5 messenger RNA and protein expression may be higher in the jejunum than in the ileum [50, 51]. Since Tac-QD is likely absorbed more distally than Tac-BID, it is possible that the lower presystemic metabolism resulting from the lack of CYP3A5 expression has more influence on Tac-BID compared to Tac-QD. To date, three studies have shown the controversial impact of *CYP3A5* polymorphisms on Tac PK when converting from Tac-BID to Tac-QD in stable renal transplant recipients [23, 49, 52].

Our study, like others, failed to demonstrate an association between Tac PK for both formulations and *MDR1* genotypes [16, 17, 19, 23].

## Conclusions

We demonstrated that Tac-BID and Tac-QD are bioequivalent in pediatric kidney recipients. The question still remains whether the definition of bioequivalence is relevant in clinical practice, in order to evaluate narrow therapeutic index drugs. In fact, a decrease in Tac exposure was demonstrated in our study population after a 1:1 (mg:mg) conversion, requiring closer pharmacokinetic monitoring during the process. The Tac-QD formulation was associated with a lower impact of *CYP3A5* polymorphisms on Tac PK parameters. Development of sampling strategies to estimate Tac-QD AUC_0–24_ may be helpful to clinicians to optimize monitoring after conversion from Tac-BID to Tac-QD. Studies to evaluate long-term adherence to this new formulation and to other immunosuppressive drugs after conversion are necessary.

## Abbreviations

AUC: Area under the concentration-time curve
C_min_: Minimum whole-blood concentration
C_max_: Maximum whole-blood concentration
Tac-QD: Once-daily tacrolimus
Tac-BID: Twice-daily tacrolimus
PK: Pharmacokinetic
Tac: Tacrolimus
t_max_: Time to achieve maximum whole-blood concentration
